# Retraction: The Relationship between Serum Insulin-Like Growth Factor I Levels and Ischemic Stroke Risk

**DOI:** 10.1371/journal.pone.0266411

**Published:** 2022-03-30

**Authors:** The PLOS ONE Editors

Following the publication of this article [1], similarities were noted between this article and manuscripts submitted by other research groups, including [2–5].

The corresponding author informed the journal that a third-party company provided assistance with language translation. They stated that similarities in text between this article [1] and others may have occurred because the authors referred to the writing methods of previous publications while drafting the manuscript.

The corresponding author also stated that the raw data underlying the results in this article [1] and the ethics approval documentation are no longer available.

The author’s comments did not resolve the concerns, which call into question the validity and provenance of the reported results and the adherence of this article to the *PLOS* Authorship policy. In light of these issues, the *PLOS ONE* Editors retract this article [1].

XD agreed with the retraction and apologized for the issues with the published article. GC, XFJ, DBT and YXW either did not respond directly or could not be reached.
